# Beyond the fingertips: imagining haptic technologies for a deafblind future

**DOI:** 10.1136/medhum-2024-013025

**Published:** 2024-11-20

**Authors:** Russ Palmer, Riitta Lahtinen, Raymond Holt

**Affiliations:** 1 Andante Research Group, University of Tampere, Tampere, Finland; 2 Mechanical Engineering, Faculty of Engineering and Physical Sciences, University of Leeds, Leeds, UK

**Keywords:** medical humanities, science communication, exhibitions

## Abstract

In this paper, we imagine how future technologies could support people who have severe hearing and visual impairment or a deafblind condition. Much effort has gone into assistive technologies to improve access for people with visual or hearing impairments, and while some of these systems will work for people with dual sensory loss, this is not always the case. Fewer systems have been developed specifically for this group. To this end, we imagine what technologies might look like in the future if they were designed specifically for people with dual sensory impairment, based on the experiences of two of the authors in accessing various displays and events related to space and astronomy. Dual sensory loss can cover a very wide range of situations, and the precise history of each individual will have a strong effect on how they use residual senses and technologies. We therefore start by reviewing literature on deafblindness, looking at current efforts to make museums accessible to people with vision and hearing impairments and social-haptic communication, a method of augmenting vision and hearing with touch signals that has developed from the deafblind community. We move on to consider three case studies, each representing a different situation: the Rocket Garden at Kennedy Space Centre; visits to observatories to view constellations and planets and engagement with the livestreamed launch of the Mars 2020 mission. For each case study, we consider the challenges faced, and the way existing technologies have been adapted or new strategies improvised to provide access to these situations. We finish by considering where these technologies might usefully go in the future—we set out some desired characteristics for future technologies, imagine some technologies for the future and how these might have been applied to the three case studies.

## Introduction

 Dual sensory loss refers to the combined loss of vision and hearing. It is sometimes termed deafblindness, although it does not necessarily indicate a total loss of these senses. Many people categorised as deafblind retain some residual sight and hearing. How much impairment is necessary to be categorised as deafblind varies from country to country and even between studies (Ask Larsen and Damen 2014). The Nordic Definition of Deafblindness (https://nordicwelfare.org/wp-content/uploads/2018/03/nordic-definition-of-deafblindness.pdf) is helpful, stating that ‘[d]eafblindness is a combined vision and hearing impairment of such severity that it is hard for the impaired senses to compensate for each other’. This makes deafblindness category in its own right, presenting challenges that are different from those faced with only visual *or* hearing impairments.

There are many causes of deafblindness, and the way in which sight and hearing loss develop has a huge impact on an individual’s experience (Swann 2010). Someone with congenital deafblindness; someone who is congenitally deaf and later suffers visual impairments; someone who is congenitally blind and later experiences hearing impairments; someone who experiences progressive visual and hearing impairments due to conditions such as Usher syndrome and someone who experiences dual sensory loss in later life will all have very different experiences and approach the world in very different ways.

This presents a particular challenge for them, as assistive technology is often designed for people with either hearing *or* visual impairment, but rarely both. While devices such as hearing aids, cochlear implants, white canes and accessibility features on computers and mobile phones can be hugely beneficial to people with deafblindness, the failure to accommodate the combined loss of senses is a leading cause of abandonment of assistive technology in people who are deafblind (Wittich *et al* 2021).

What would technologies specifically aimed at people with dual sensory loss be like? Haptics—communication through the sense of touch—is one potential avenue for providing information in a way that is usable by people with dual sensory impairments. Haptics are already in use for communicating with this client group−for example, tactile sign languages, deafblind fingerspelling or ProTactile (Van Der Mark 2023), or the use of braille or tactile images−but these are not catch-all solutions: the sense of touch can be impaired in older people, and not everyone has learnt tactile sign languages or braille. Nor is it straightforward to convert everything into a tactile form (Pakenaite *et al* 2024). The speed of the tactile system is much lower than the speed of auditory or visual processing: and both auditory and visual processing tend to be integrative, pulling together a variety of points of information very quickly. Exploration through the sense of touch tends to take much smaller point samples of an environment, meaning that it can be difficult to interpret cues that in vision or hearing would be simple (Klatzky and Lederman 2011). Tactile images, for example, are difficult to interpret without appropriate context. There is a risk of ‘lowest common denominator’ design—restricting interaction and communication to what can be readily delivered through the sense of touch, when it is better to use haptics to augment the technologies and residual senses people with dual sensory loss already use.

There is also a significant risk in using technology to replace human contact. While reducing dependence on others can be beneficial, particularly when assistance is not readily available, deafblindness can be extremely socially isolating, and there is a danger that technology can cut off one of the few human contacts a person has. In this paper, we argue that new technologies must augment, rather than replace, existing devices and social relationships.

We will review how haptics and tactile information can augment visual and auditory information for deafblind people to improve access to information and experience using three case studies based on the experiences of one of the authors (Russ) who is deafblind using two cochlear implants and another author (Riitta) who acted as a describer during these case studies. The case studies will focus on the theme of space and astronomy, as these are of particular interest to Russ, and particularly to cases where the objects of interest cannot be directly felt. The first case discusses a visit to the Kennedy Space Centre where the artefacts on display are immense rockets and space capsules that cannot be handled; the second considers trips to observatories, where the information of interest is the position of stars and planets and the third considers participation in a live rocket launch, viewed over the internet. It is important to recognise that these represent the experiences of one deafblind person, and will not apply to every person with deafblindness, but act as a starting point for discussion. Before looking at these specific case studies, however, we will first examine the current approaches used to convey information to people with impaired vision or hearing in contexts such as museums, and social-haptic communication methods developed specifically for people with dual sensory impairments.

## Information access and social-haptic communication

Given the significance of residual sight and hearing for people with dual sensory loss, they may find many devices intended to aid people with visual impairment or hearing loss alone to be very useful. For example, hearing aids and cochlear implants can allow access to audio assistance intended for the visually impaired, while braille and tactile images can help provide information that a person cannot perceive visually.

Nevertheless, access to these is not guaranteed—someone who only becomes visually impaired in later life is unlikely to have learnt braille, for example. Relative to visual and hearing impairments, there is significantly less research into technology that supports the particular needs of people with dual sensory impairments (Dyzel *et al* 2020). Failing to consider these particular needs is a significant cause of device abandonment (Wittich *et al* 2021).

The natural place to consider accessibility and access to information, particularly in light of the case studies we have selected, is museums. Museums aim to inform visitors about a given subject, such as history or science, and there have been some significant efforts made in recent years to improve access to museums. Here, we find a similar pattern to that in technology more generally: there has been work on improving access for people with visual or hearing impairments, but rarely both. Research on improving access to deaf and hearing-impaired individuals emphasises the significance of signed tours to supplement the static written materials provided in museums (Eikelenboom *et al* 2019; Martins 2016). Kim *et al* (2023) note that one challenge here is having someone who is both fluent in signing and knowledgeable in the subject area, and explore the idea of interactive prototypes, where interactive text is provided, and users can provide queries. While some people who are deafblind may have sufficient residual vision to access these resources, this is a problem for those with more significant vision loss.

Museums are largely a visual experience, with objects displayed in cabinets, often with accompanying signage: research has therefore tended to focus on making museums accessible to the visually impaired. Vaz *et al* (2021) and Fortuna *et al* (2023) examine the challenges faced by visually impaired visitors in Portuguese museums and the Grand Rapids Public Museum in Michigan, USA. They highlight the importance of residual senses, the difference between those who acquired visual impairments early in life and those who acquired them later, the importance of providing touch objects and providing audio to supplement these, and the importance of staff training to provide support and assistance.

Vaz *et al* (2020) provide a helpful overview of the literature on the barriers faced by visually impaired people when visiting museums, along with examples of assistive technologies that may be helpful. They note that touch tours remain the main method for visually impaired people to access museums, but that tactile information alone is not sufficient and that contextual information must be provided to aid understanding (Hayhoe 2017). They identify four categories of assistive technologies that can assist visually impaired visitors in museum visits: haptic interfaces (allowing touch tours of virtual objects); interactive touch replicas (where sensors are embedded in a replica of a three-dimensional (3D) object so that auditory contextual information can be provided as they explore the object by touch (such as Tooteko—see D’Agnano *et al* 2015); gesture-based reliefs (similar to the interactive touch replicas, but where a tactile image of an object is provided, and a camera tracks the finger-based exploration) and assisted navigation (that helps visually impaired individuals navigate the museum, rather than interact with the exhibits).

Along the same line as the interactive touch replicas and gesture-based reliefs described by Vaz *et al*, there has been work on the concept of ‘smart exhibits’ (Cavazos Quero *et al* 2021; [Bibr R18][Bibr R17]; Vassilakis *et al* 2018), which provide responsive interaction, such as on-demand audio and multisensory experiences. Montusiewicz *et al* (2022) provide a methodology for developing 3D models with integrated braille from scans of original artefacts. Nofal *et al* (2023) provide an overview of 24 systems that aim to augment physical replicas in museums with interactive digital features.

All these papers treat hearing and visual impairment as independent. We have not been able to find any works specifically on museum accessibility for those with dual sensory impairments. This is not entirely surprising: deafblindness is a rarer condition than either hearing or visual impairment alone, and the needs of individuals with dual sensory impairments can vary greatly. Even among those who are deafblind, technologies such as hearing aids or cochlear implants can access the audio features that make some of the proposals for visually impaired individuals accessible, although they may also require supplementary information to help make things clear. Others will not be able to access these resources at all, requiring more complex approaches.

One approach that has been developed to assist in communication and orientation for people who are deafblind is social-haptic communication, originally developed by Lahtinen and Palmer (2013) to augment speech or signing with additional information by delivering drawing touch messages called ‘haptices’ onto the body. Haptices can be straightforward linguistic messages, such as ‘drink’, ‘laughter’, ‘angry’ and are often used to convey contextual information while the recipient is speaking or signing. They are typically delivered to the back but can also be delivered on other parts of the body, such as the arm, shoulder or leg. These can also be used for environmental description (Lahtinen, Palmer, and Lahtinen 2010), such as room layouts, or the position of people or objects of interest, allowing a deafblind person to build a mental image of where things are in relation to them. This is particularly helpful in the context of museums and cultural exhibits, allowing the description of images and objects and can augment narrated descriptions or touch tours of objects.

On the topic of space and the solar system, there have been projects such as the Tactile Universe Project (Bonne *et al* 2018), which uses 3D printing to create tactile images of planets, galaxies and solar systems. In recent years, Palmer (2021) has also proposed haptices for conveying features of the solar system through social-haptic communication known as astro-haptices (Palmer and Ojala 2023).

These represent a variety of methods for conveying information to people with sensory impairments, although only social-haptic communication is specifically developed for those with dual sensory impairment. Hearing aids or residual vision can make other methods (such as smart exhibits) accessible, and much can be done to ensure that people can make the most of their residual senses. In the upcoming case studies, we will consider some of the challenges faced by a deafblind individual, and how they are addressed.

## Case studies

In this section, we present three case studies representing situations in which one of the authors, who is deafblind using two cochlear implants, has been engaging in activities related to his interest in space. Each represents a different context and different challenges. The first is a trip to a museum, the Kennedy Space Centre, and particularly the experience of rockets and capsules; the second is based on two trips to observatories, to observe constellations and the planet Jupiter and the final one is based on experiencing a livestream of a rocket launch. Each represents different challenges, and we consider how these have been addressed, and then reflect on lessons that can be learnt for the future.

### Kennedy Space Centre

One of the major attractions of the Kennedy Space Centre in Orlando (Florida, USA) is a ‘rocket garden’: an outdoor collection of rockets from the earliest stages of the space programme onwards.

While these enormous rockets were a critical—and highly visible—part of space missions, the crew experienced these missions through a cramped crew compartment: a far cry from the vast scale of the rockets carrying them! Within the Visitor Complex, there were a number of these crew compartments from the National Aeronautics and Space Administration (NASA) missions, so visitors could see the quarters in which the astronauts had to operate.

Russ already had a substantial knowledge of these rockets and the space programme, so the goal was not to convey historical information but the experience of being in the presence of these historic objects. The Kennedy Space Centre website (accessed 27 June 2024) describes it as: ‘[a] visit around the Rocket Garden is like taking a stroll among titans’. That sense of presence was an important part of the experience for Russ.

#### Challenges and solutions

The rocket garden is designed on a ‘look, but do not touch’ basis, making it inaccessible to those without vision. Guided tours are provided, but these are aimed at conveying the historical and technical background which Russ already knew. The scale of the rockets make a touch tour impractical: there is no possibility of taking in a whole rocket through a few touches (the smallest, Juno 1, is 21.7 m tall; the largest, Saturn 1B, is so tall at 68 m that it has to be laid on its side).

Several solutions were adopted to address this. First, Russ and Riitta brought seven scale models of the rockets with them, that Russ already owned and was familiar with by touch. This had two benefits. Riitta could identify which model went with which rocket by sight, and by passing the model to Russ, allow him to know which rocket she was referring to. It also allowed her to highlight particular features to him, by pointing them out on the models, allowing Russ to know what she was referring to.

To address the issue of scale, they paced out the length of the Saturn 1B together, presenting the height kinesthetically, and giving Russ a sense of the scale relative to his own body. This was then used as a benchmark, with Riitta drawing the relative heights of the other rockets (which were positioned vertically) on Russ’s back, so that he could appreciate their scales without the need to pace out each one in turn.

Additionally, Riitta made use of Russ’s white cane to point out the location of features from the models on the actual rocket. The benefit of doing this was that it again gave a kinesthetic sense of relative positions of items and allowed Russ to make use of his residual vision, as the cane was much easier to see and follow than a finger.

Both these strategies provided interaction with the environment (walking along and pointing to) that could be experienced bodily, without the need to touch the actual exhibits. This was important, because it helped to convey the sense of presence to Russ in a way that a purely narrative description would not.

Access to the crew modules was a different matter: there were huge numbers of controls, and space inside was very limited. Whereas sighted visitors could look into the capsules and quickly see the complex and claustrophobic environment, this is not an option for those with visual impairments, and a verbal description of the layout cannot convey the experience properly. Here, however, special arrangements were made for Russ to enter the capsule himself and experience the compartment through tactile exploration.

This presented its own challenges: the compartments were cramped and narrow, making it impossible for an assistant to accompany Russ into the compartment, and making it difficult for him to navigate his way in. In the end, Riitta had to use a personal radio microphone system to direct Russ to help him climb up the access ladder, and then orient himself into one of the seats. The entrance hatch presented a particular problem, as there was some risk of striking his head, so Russ had to carefully use his hands to explore the opening and understand its dimensions and its position relative to his body.

#### Observations

This case study highlights several things about the museum.

First, *the importance of the museum as an emotional experience, rather than just a source of information*. For Russ, who was already well-versed in the background of space exploration, the main value of the trip lay in the experience—being in proximity to the rockets and capsules that had been part of the space race. The scale of the rockets and the cramped conditions of the crew capsules provided a tangible sense of the challenges faced by the astronauts. It was a way to share something of the experience of those who had actually travelled into space. This could not be conveyed by audio descriptions alone: these make the information accessible, but not the experience. It is, of course, worth noting that not every visitor will necessarily share this desire—for some, it may be that the information and learning more about the space race and NASA programmes is the most important part of the visit. Still, there is a reason that the Space Centre displays actual rockets and crew capsules rather than reduced scale models, to give a sense of the scale of the work, that cannot be conveyed by words alone.

Second, *the significance of prior knowledge* that allows an individual to interpret the information being supplied. In this case, Russ had a great deal of prior knowledge to tap into, which was a great benefit in helping him to interpret the information presented to him; conversely, Riitta did not have the same level of knowledge, so a way was needed to communicate between them that would enable Riitta to highlight features that Russ would recognise and know.

Third, it is worth noting the significance of *hearing aids and cochlear implants* that enabled verbal communication between Russ and Riitta, meaning that they did not need to rely on purely social-haptic communication. Indeed, it is worth noting the extent to which Russ made use of his residual vision to access information.

Fourth, it highlights the importance of *skilled assistance*: the presence of someone who could act as a bridge between Russ and the environment to direct his residual senses to important features and provide communication and clarification. This required a degree of familiarity with Russ and social-haptic communication techniques, as well as other methods he was using (block letters, haptic exploration) that go beyond just giving a standard guided tour commentary.

### Visits to observatories

Our second case study concerns visits to two observatories—one in Finland, and one in Denmark—which we have grouped together because they represent similar situations. The first visit (Finland) represented an opportunity to speak with observatory staff and find out more about constellations visible through the telescope. The second visit (Denmark) provided an opportunity to look at the moons of Jupiter, which are not visible to the naked eye. Both observatories provided an opportunity for visitors to look at stars and planets through a powerful telescope—but this is a primarily visual experience, which is not easily accessible to people who are visually impaired. The opportunity to see the moons of Jupiter was particularly significant for Russ, as he still had a degree of residual vision, although with a very narrow visual field, and he wanted to make the most of the opportunity to see the moons for himself while he still had the chance. In this particular case, Russ’s prior knowledge was of less value—knowing about Jupiter and its moons or about the names of constellations does not help to capture the experience of seeing them, or the difference between what can be seen by the naked eye and what can be seen through a much more powerful telescope.

#### Challenges and solutions

Each visit presented particular challenges. The first one was to have a skilled guide and describer who had some knowledge of astronomy and planets. The second issue in both cases was Russ’s restricted field of vision. This mean that, rather than being able to take in the view from the telescope’s viewfinder as a whole and then shifting attention to parts of interest, Russ could only view a small portion of it at a time. This made it, first, impossible to view a constellation as a whole, and second, very difficult to locate where to look in order to take in different parts of the view. This was compounded by the fact that, while those with Russ could tell where the telescope was pointing, it was impossible for them to know which parts of its view he was looking at. As a result, they could not give directions to help him.

Russ’s further challenge is that a degree of knowledge is required to interpret what is being seen. Without this, it is difficult to offer richer descriptions than ‘there are a lot of stars’ or ‘there is a big bright dot surrounded by smaller dots’. This was not something that Riitta had, which meant relying on the expertise of other describers who were available.

**Figure 1:**
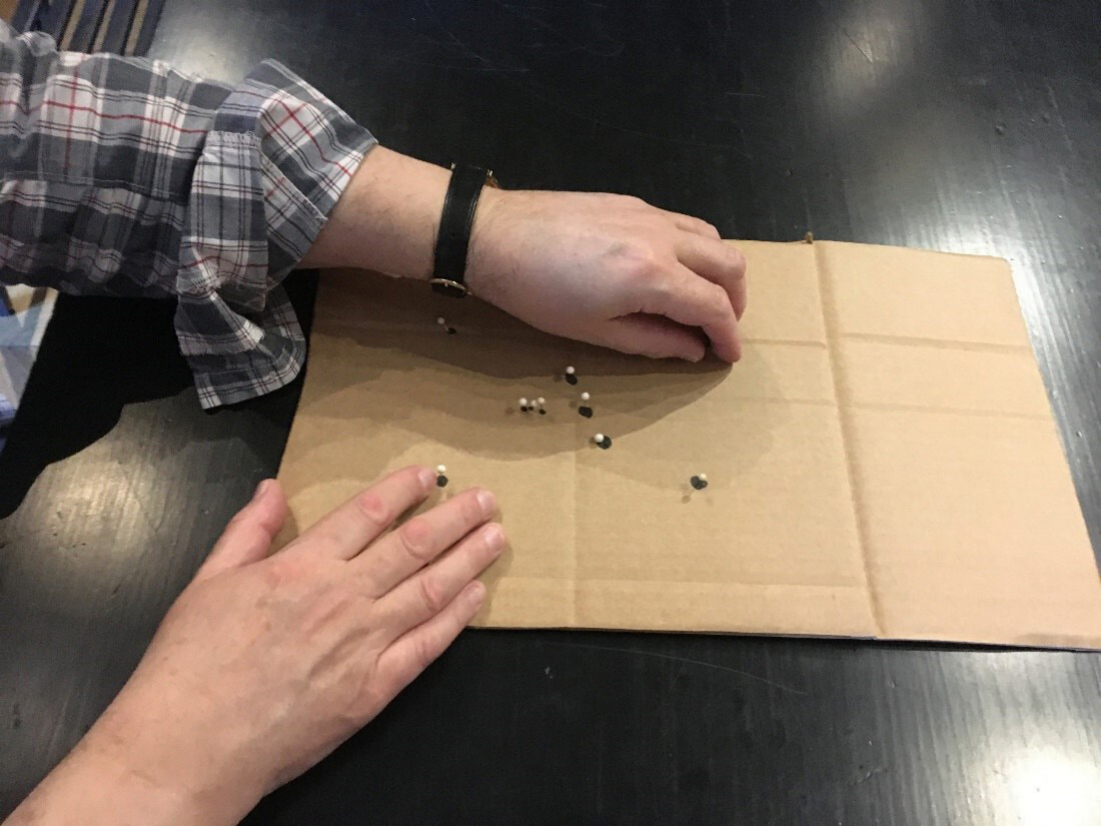
Author A interacting with an improvised tactile diagram of a constellation.

In Finland, where the challenge was to present constellations, the staff at the observatory came up with the idea of using pins and cardboard as shown in figure 1. In this way, they could lay out the significant stars in a constellation, and allow Russ to feel them, and get a sense of their overall shape. In this way, the staff could see which parts of the tactile image Russ was interacting with, and thereby give him advice on what he was touching and how to get to other parts of the constellation. This also allowed Russ to pose questions to the staff, stating his interpretations and checking whether he was correct.

In principle, a similar approach could be used with the moons of Jupiter, laying out where they were in relation to the planet, but this fails to capture Russ’s main goal in the visit: to see the moons of Jupiter with his own eyes. As with the visit to the Kennedy Space Centre—and unlike the trip in Finland focused on constellations—the goal was not to obtain information, but to have a specific experience. Just creating a tactile model to explain where the moons were was not the same as actually seeing them.

To address this, a finder scope (a smaller scope attached to the telescope that provides less magnification but a wider field of view, to be able to aim the main telescope in the right general direction before fine-tuning its positioning) was used, which allowed another person to get some idea of the view Russ was receiving, and then using social-haptic communication and environmental description strategies to convey information. For example, using a clock face to describe where one of the moons was in relation to Jupiter (‘at five o’clock’), so Russ would know which way to look from the main planet. Russ also had to describe what he was seeing in order for the others to give him directions. Additionally, giving information such as highlighting the stripes of Jupiter by drawing on his back so Russ had some idea of what to look for.

A second assistant was required, since it would be impossible for someone to look through the finder scope and draw haptices onto Russ’s back at the same time. It helped that the second describer was knowledgeable in astronomy and planets, so she knew the important features of Jupiter and its moons and where to look for them and was able to provide appropriate descriptions.

#### Observations

We see once again the importance of the *emotional experience rather than just information* to events: the significance of A seeing Jupiter himself, rather than just being told about it. The Finland case provides a contrast: there, the focus was very much on information, and this allowed a different strategy, focused on conversation and haptic guides.

Russ’s own prior knowledge was less important, but *interactions with knowledgeable people* were more important. It is worth noting that this was a two-way interaction, Russ asking questions and receiving answers, or being advised on which parts of a constellation he might be touching when using the tactile images. This is different from a prerecorded audio description, which—while potentially useful—does not provide the detailed real-time answers to support haptic exploration and active interaction.

There is also the requirement to communicate direction and guide Russ towards features of interest. This was more challenging when Russ was using a telescope to view the moons of Jupiter: whereas with the tactile images it was very clear to anyone assisting him which part of the image he was experiencing at a given time, it was impossible to tell where he was looking. This required a different strategy, of offering orienting information.

We again see the importance of residual senses, both sight and vision, and the importance of technologies such as cochlear implants or hearing aids to enable Russ to make the most of them and communicate with those around him. Again, though, we see that they are not a perfect substitute—there is a need for social-haptics and haptic exploration to augment the information received.

### Mars 2020 rocket launch

Our third case study concerns the launch of NASA’s Mars 2020 mission. The mission is one of a series to Mars run by NASA, with the goal of searching for signs of microbial life, investigate the possible habitability of Mars in the future and to collect rock samples with the hope that later missions will be able to collect them and return them to Earth. The Mars 2020 mission comprises the rover *Perseverance* and the mini-helicopter *Ingenuity,* which were launched on 30 July 2020 aboard the Atlas V-541 rocket. *Ingenuity* was of particular note, as it represented the first powered flight on mars. The mission was not a singular event, but an ongoing process: the flight to Mars would take over 6 months; the mission on Mars was due to last at least 687 Earth days (and is in fact still in progress at the time of writing) and years of work had gone into the mission prior to the launch. Nevertheless, the launch itself marked a major milestone in the project, an important moment for those following the mission and was livestreamed via NASA’s website. Watching live was an important part of the experience for Russ: the fact that the launch was happening in real time created a sense of connection to the event and to others around the world who were also watching live that would not have been possible if it had been watched after the fact.

#### Challenges and solutions

The livestream presented accessibility challenges for Russ. While Russ could hear the commentary through his cochlear implants, it was not always clear—presenters sometimes spoke quickly to fit with events on the screen, and broadcasts from the control room were of lower quality than broadcasts from the studio. Being a live broadcast, there was no possibility of rewinding and relistening. Also, the visual elements were an important part of the livestream and were completely inaccessible to Russ.

Much of this could be addressed by having a describer in the room with Russ, who could contextualise information through haptices, touch messages: in this case, Riitta. However, to understand what was happening, the describer needed a level of familiarity with the subject matter that Riitta lacked. To address this, a second describer with expert knowledge of the subject joined via a mobile call to provide additional commentary. This included details about different rocket stages detaching, or propellants coming out of the rocket. In this way, Riitta was able to listen to both the live commentary and the additional explanations from the other describer and provide important haptices onto Russ’s back.

**Figure 2:**
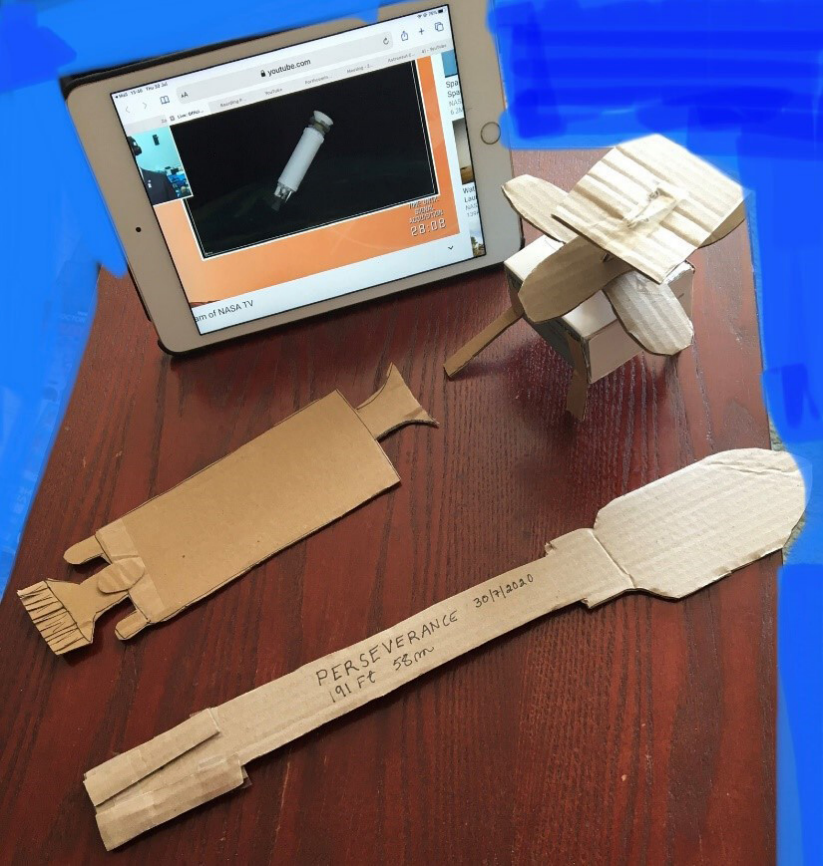
Home-made tactile props for the Mars 2020 launch.

A particular issue was that the prior knowledge that served Russ well in the first case study was actively unhelpful here. The Atlas V rockets are very different in appearance to the Saturn 1B or Mercury Redstone rockets that Russ knew. Also, the mini-helicopter *Ingenuity* did not resemble the conventional helicopters that Russ retained a visual memory of. It was necessary to find ways to convey this information to him. For this purpose, simple cardboard silhouettes were cut out, as shown in figure 2, so that Russ could feel the differences in shape.

#### Observations

Yet again, we see the *significance of emotional experience rather than transfer of knowledge*. Watching live was an important part of the experience, knowing that events were happening in real time, and not prerecorded. This had immediate impact on the level of preparation that could be made. While it was possible to make some simple models in advance by Riitta and the other describer researching the topic, there would always be some elements that had to be addressed as they happened.

The importance of *knowledgeable describers* was again highlighted: knowledgeable in both the subject and in communicating with Russ. As before, this required different describers in different roles.

In this case, we note that *prior knowledge* can be actively unhelpful and sometimes needs to be corrected.

And for the third time, we see the benefit of *tactile models*, even when made in low fidelity, to enable understanding of items in a way that would be very difficult to convey using words.

## Discussion

Several common themes emerge across these case studies. The first is the importance of *sharing an experience* rather than merely acquiring knowledge: being in the presence of immense space rockets; experiencing the cramped quarters of crew capsules; seeing the moons of Jupiter and sharing the launch of the Mars 2020 mission with fellow enthusiasts around the world. This creates the need to engage with things in real time, not after the fact, limiting how much preparation can be done in terms of looking up details or concepts or preparing tactile materials. This also means that simple information transfer is not sufficient and engaging the whole body in the process becomes important: feeling the constraints of the capsule, pacing out the size of the rockets to give a tangible sense of scale.

We also see the benefits of *tactile resources*, such as models of space rockets and tactile images, even in a basic form. These provide a way for assistants to tap into Russ’s prior knowledge when the name of a feature is not known; and as way of conveying new information to him that has a shape or spatial component (in the case of the constellations and the Mars 2020 launch). It is worth noting, however, that producing even low-fidelity tactile resources is labour intensive, and not guaranteed to happen even if there is time to prepare in advance.

To provide assistance to Russ, both subject knowledge and communication knowledge are required, and these rarely exist in the same person, sometimes necessitating an additional assistant. The ability to adapt to an individual’s prior knowledge also becomes important: this is a rich resource that can be called on if managed correctly but may also end up being a problem if things have changed or are slightly different from what is expected.

We also see the importance of the residual senses, and assistive technologies that help to make the most of them. Nevertheless, information often needs reinforcing, provided in multiple modalities in order to make the most of them.

There is also the importance of *interactivity*: instructions and guidance that react to what a person is doing, allowing the person to set the pace and control the direction of discussion, rather than obliging them to passively receive information. Similarly, allowing an individual to ask questions is important, letting them clarify matters before moving on. It is worth noting that these are all likely to be different for different people, depending on their personal preferences and experiences, and the particular situation they find themselves in.

Then there is the question of *social* connections. All of the contexts we have described have relied on having at least one describer present, and sometimes two. This can be inconvenient—it is not always possible to have that much assistance on hand. Mobile phones have made it easier to tap into domain expertise when the individual is not actually able to be present in person (as was the case with the rocket launch case study), but they cannot substitute for the tactile elements. If Russ had been at home alone when the launch took place, he would not have been able to engage with it in anything like the same way: the experience might be synchronous, but it would be significantly impoverished, missing out all the visual elements. One of the conveniences of technology is that it allows access when an assistant cannot be available. This offers more independence for the deafblind person and allows them to do things they would otherwise not be able to do. However, there is a counterpoint to this: deafblindness can be extremely isolating. Removing the few points of human contact that are possible and replacing them with technology serves to compound it: ironically, technology can therefore make activities more inclusive and more isolating. Russ could have had an interactive guide of some form that would allow him to explore the Rocket Garden alone, or watch the livestream with additional commentary, or social-haptics provided by a device. This would make it easier for him to access, and would ensure that he could experience the launch live even if assistance had not been available. On the other hand, there is a risk that such events could become extremely lonely, and when these events are being attended to as a hobby, there is a desire to share them with other people, rather than experiencing them alone.

One of the important features of deafblindness is the need to obtain information from multiple senses, and to triangulate between these sources—sometimes over a period of a time. For example, vision tends to be holistic—a lot of information can be processed very quickly, with the eyes focusing on and moving between important features very quickly. Furthermore, assuming that an object or a sign or an image remains in place, it can be explored visually at leisure. Audio is more difficult to navigate, because any given sound is by its nature instantaneous: this is a particular problem where hearing is impaired, and parts of an audio signal may be missed. Even if an audio signal (eg, a description of an object) is repeated, this can take time and requires remembering which bit was missed. This is less of a problem when interacting with another person, who can repeat specific phrases or clarify their meaning on request, but navigating prerecorded audio is much more challenging, taking attention away from the current situation in order to find the required information.

Tactile exploration of an object has the benefit of being persistent in the same way that vision of the object is, but touch can only capture a small portion of an object at any given time. This means that more time is required to explore, remembering what has come before, and trying to work out where to explore next in order to build up a mental image of an object. Important features may be missed completely, or difficult to relocate unless there is some way of guiding the exploration.

The final thing that we see from these case studies is that in every case, solutions were *assembled*. There was never a single pre-existing device or solution that was used: rather, Russ was enabled through a combination of people (most notably Riitta), existing assistive devices (most notably his cochlear implants), improvised tactile resources (purchased models; pins stuck in card and simple cardboard cutouts) and a strategy of social haptic communication. This is critical to imagining how technologies might work in the future: to talk of a technology or a solution is wrong. Any solution to the problem of accessing or exploring situations will necessarily rely on a patchwork of sociotechnical elements, which will differ in every situation.

It is vital that designers and technologists bear this in mind when developing new devices. It is not feasible to create a bespoke device for each person for each situation, but in making devices that apply more generally, there is a risk of forcing users down a particular route and constraining their ability to assemble solutions themselves. New technologies should not aim to solve a problem, but to enable individuals to assemble their own solutions.

What, then, might a useful technology for Russ be? Are we imagining a ‘cochlear implant for the eyes’ that would allow him to access visual information through a camera and a brain implant? Or does this mean we are just imagining ‘fixing’ or ‘normalising’ Russ? While a cochlear implant offers access to hearing, it is not perfect. Similarly, while a device that offers some access to vision may be useful, if it is grainy, low resolution or limited by natural light or noisy visual environments, additional support will still be required. If our imagining of technologies for deafblind people in the future is that technology would make them ‘not deafblind’, then we are imagining a future with no deafblindness, which is not the scope of this paper.

Any new technology should offer Russ options—rather than trying to do everything, it should be part of a toolset that can be called on to address a given situation. Expanding the palette of options, rather than narrowing them down: but this means taking account of a potentially huge range of situations in which a technology might be used. This brings out the central tension in imagining future technologies: any technology only useful to Russ would be financially unviable, but no single device or technology will be useful to every person with deafblindness. In the next section, therefore, we will start by imagining technologies specific to Russ, then consider how they might apply more broadly.

To summarise, we propose that technology designed to support Russ in the absence of domain experts or interpreters should:

1. Enable transfer of information in real time, to enable simultaneous participation in events;
2. Convey emotional content, not just the factual and informational content;
3. Make the most of residual senses, and provide a multimodal experience to make it as accessible as possible;
4. Be interactive—allowing the user to seek and clarify information, and assemble the information available to them in their own time, rather than just providing it in a continuous stream;
5. Allow the user to make the most of their prior knowledge, and provide new knowledge when needed;
6. Supplement, rather than replace, existing human interaction. For example, by facilitating shared social experiences that would not otherwise be possible;
7. Guide the user to information they are interested in and inform them when they have missed important information.

## Imagining technologies for the future

We can imagine two situations in which technology would be beneficial to Russ in scenarios like those presented in our case studies. The first is when he does not have access to a domain expert, and therefore needs to ask questions and clarify matters. The second is when he does not have access to an interpreter such as Riitta, and therefore requires support in communication and understanding what is going on.

Smart exhibits might help to address the former case, but we are losing the interactive conversation of having a human domain expert present, where questions can be posed, answered and clarifications made as needed. Also, smart exhibits make sense in a museum context—where it is expected that each exhibit will remain in place for some time, and large numbers of people will interact with it over time. In the home, such as in the Mars 2020 launch, the effort involved in creating one would be significant. In the latter case, where no describer is present, the ability to interact with the environment proprioceptively or to receive social-haptic communication signs through a device would be useful. We will therefore imagine two corresponding technologies: self-narrating tactile resources and mediated social-haptic communication.

### Self-narrating tactile resources

In self-narrating tactile resources, we imagine a tactile resource where a user can ‘pull’ information as they explore, rather than having it ‘pushed’ at them. As they move their fingers across the object, they could request different sorts of information—from a simple label (in the case of the rocket, eg: ‘payload’, ‘fuel tank’, ‘booster’); to more detailed descriptions of that part’s appearance or purpose. In a museum situation, this would go some way towards accommodating different levels of prior knowledge, allowing a user to orient themselves without having to listen to a lot of background information. The methods for tracking the area explored are already addressed by the smart exhibits reviewed in the ‘Information access and social-haptic communication’ section. How to control what information is requested is trickier. With two levels of detail, dwelling with the finger for a short time might be sufficient to trigger an audio label about the point being touched, while dwelling for a longer period might generate a more detailed description. If we wanted to provide different types of information, then some other system would be required—multiple buttons are one option, but this precludes using two hands to explore the resource. A gestural interface might be another, where tracing different patterns over a hotspot request different types of information. Alternatively, we might imagine the use of speech recognition and language models to allow users to actually converse with the system and ask about what they are touching.

In terms of our case studies, this approach is most useful in the Mars 2020 launch, for exploring the Atlas V rocket; and *Ingenuity* min-helicopter; or the Capsules at the Kennedy Space Centre, where simple explanations that name a part or its function are most helpful. It is less useful in the Rocket Garden, where parts of the rockets were already known, and the value of the models was for communication between Russ and Riitta; or in the case of the constellations, where the names of individual stars were less useful than the overall shape of the constellation and the positions of the stars making it up.

The problem is that these would be extremely complex to set up and could not be improvised in the same way as the low-fidelity models used in the observatories and Mars 2020 case studies. They lend themselves to fixed museum exhibits where they can be part of a strategy offering access to larger numbers of visually impaired individuals, rather than as part of a toolkit for a single individual for one experience. We might imagine an ‘instructables’ approach, an archive where items could be 3D printed, and assembled at home with preloaded programmes, but this requires significant technical ability and swaps out one expert (domain) for another (technical). Of course, the ideal would be some fully haptic 3D display that could render and narrate any model downloaded from an internet archive, so that no assembly was required, but sadly, this remains a pipe dream.

### Mediated haptic communication

The live narrative and social-haptic communication presented by the two assistants in the Mars 2020 system are the most difficult aspects to replace technologically. But in an ideal world, the system would recognise in real time what was happening and supplement the audio with additional haptices to convey the information. This would then require a technology for delivering the haptices, which is a challenge in itself, but would also open up the possibilities of remote haptic communication between Russ and Riitta when they are not able to be together; or for someone else to provide similar support in other social situations.

The critical part of this is the device for conveying haptices. Efforts have been made to recreate haptices through an array of vibrotactile motors on the back (Plaisier and Kappers 2021), and vibrotactile vests (such as the bHaptics TactSuit X40 or Actronika’s Skinetic) are commercially available. Nevertheless, it is unlikely that a vibrotactile actuator can offer the same level of fidelity of signal as direct human contact, and they also cannot reach parts of the body such as the arms or hands that are sometimes used as recipients in social-haptic communication. Also, such vests are bulky, warm and conspicuous. They might be reasonable in the home, but then alternative approaches such as mounting actuators on a chair or cushion might be better.

If we free ourselves from practical constraints in our imaginings, our idea would be a light, wearable vest or cushion that could be used to deliver haptic signals to the back, directly replicating the sense of touch. We would imagine two inputs to it: one is a direct input, whereby Riitta or another interpreter could use a tablet device to draw signs that would then be sent directly to Russ’s back. This would allow for the same flexibility of communication as when they are together in person, but make it available in situations where they are physically apart. A second possibility would be having museum exhibits or videos having ‘haptic captions’, whereby haptices would be triggered at certain points of a video or certain explorations, for example, to provide context. Entering a room or exhibit, for example, Russ might be presented with a description of its layout, with accompanying haptices; or it might be possible to request size comparisons between the different rockets and have these drawn in relation to each other. Live ‘haptic captions’ would be more difficult in the Mars 2020 launch case. Here, we might have to rely on significant object recognition to automate things in real time, or on a centralised person drawing signs on a tablet that others could tune into, a little like having a live sign language interpreter. This would certainly make the launch more accessible without the need for additional assistance.

Finally, this would open up the possibility of interfacing with other devices, such as cameras and machine vision, to capture emotions, identify who is talking and indicate where the speaker is, making social situations easier for Russ when Riitta is not available. It also offers the opportunity to interface with computer vision systems like BeMyAI in a way that makes spatial elements easier to understand than in a text description alone.

### Discussion

There are many ways that technology can assist people with dual sensory impairments: cochlear implants, hearing aids and glasses open up interactions with the environment that would otherwise be inaccessible. But these do not automatically offer perfect vision or hearing, and often benefit from additional multisensory support. It is tempting to suggest that the solution to future technologies for people who are deafblind is to work purely in terms of haptics, but the sense of touch is a very limited modality, and this can also be impaired, so it is better if sensory modalities supplement, rather than replace, each other.

Does it even make sense to talk of ‘technologies for deafblindness’, when deafblindness is such a varied condition? The needs of people with congenital deafblindness are very different from those who develop deafblindness in later life. We note the significant role that Russ’s visual memories of things he had previously seen played in allowing him to understand the tactile models: the same approach would not work well for someone who had been congenitally deafblind, for example. The reality is that the most appropriate method will depend on the individual. And why focus specifically on ‘technologies for deafblindness?’ Might similar strategies not work for other groups? Certainly, multimodal communication has begun to be used with people who have dementia or learning impairments. We further need to consider the issue of transition: the major causes of deafblindness (age, Usher syndrome) are progressive. The best technologies for a given person will change over time and the process of transitioning between technologies is an important one.

There is a tension here: one reason for considering ‘technologies for deafblindness’ is that a key reason current assistive devices are rejected by people with deafblindness is that technologies designed for people with visual *or* hearing impairments do not necessarily work well for them (Wittich *et al* 2021). Yet, we also note how valuable Russ’s cochlear implant—a technology aimed at people with hearing impairments rather than specifically deafblindness—has been to him in all these case studies, precisely because it enables flexibility and relationships.

Furthermore, we might question the emphasis on technology itself. People played an important role in all three case studies: as a minimum, a describer (Riitta) who has long experience working with Russ, and in two cases domain experts. Technology was used as an intermediary to enable that communication: again, Russ’s cochlear implant and (in the case of the Mars 2020 launch, a phone) were significant in enabling these human interactions.

Also, it is worth considering the relationship between Russ and Riitta: they have a working relationship developed over decades, which cannot be quickly replicated with just any interpreter. Most people with dual sensory impairments do not have a Riitta to work closely with them. They could not access the experiences described in the same way without some additional support from either people or technology. This is another tension—not everyone has access to the sort of support that Riitta provides to Russ, but is the solution better access to people, or better access to technology? Perhaps a mix of both. In this paper, we cannot offer ‘a’ solution to this, as it is likely to be highly individualised.

What does this mean for technology development? Our core argument is that technology should not be ‘a’ solution, but expand the options available to people for solving problems. Rather than imposing solutions, new technologies should offer new options that allow people to construct their own solutions, based on their individuals.

How do we do that in practice? Perhaps even designing assistive technologies needs to come from an ‘inclusive’ approach. Inclusive design and assistive technology are not at odds, but the emphasis needs to be on enabling inclusion, rather than fixing impairments. Greater flexibility and personalisation seems important, rather than trying to find completely self-contained solutions that impose a particular way of interaction on the individual.

Finally, we need to consider not just individuals’ impairments, but their knowledge and digital literacy. To use social-haptic communication requires time and training: a device cannot just be parachuted in and deployed without appropriate training, except where someone is already familiar with haptices of this type. Similarly, any complex device requires setup that may be trivial for someone with prior experience of technology and programming, but impossible for those lacking such a background.

## Conclusions

In this paper, we have reviewed some existing approaches to making information available to people with hearing, visual and dual-sensory impairments, particularly the idea of smart exhibits in museums that provide a multisensory tactile and audio experience; and social-haptic communication that provides contextual information to supplement other sources. We have reviewed three case studies of a person with dual sensory impairments engaging with various sources of information related to space exploration. There are technologies currently available that can assist with this, and even research concepts such as smart exhibits provide some support by extending traditional tactile objects into something more interactive. However, we imagine future technology could extend this to allow proprioceptive display of information (through body position) to engage with larger objects and scenarios that cannot be made into tactile objects; through haptic communication that could supplement information with contextual details through haptices and through more interactive controls, allowing not just a display of information to be ‘pushed’ to the user, but to enable the user to ‘pull’ information, request clarification or repetition and so control the pace of exploration.

Our major observation is that ‘solutions’ in these cases are personalised, and assembled from available resources (both people and technology), and we argue that trying to develop any technology that attempts to provide a whole solution to one of these situations would necessarily be of very limited use, even to the individual it was designed for. Rather, we observe that the important things are the ability to share in experiences, to participate in real time and the significance of prior knowledge. Imagining technologies for the future, we imagine those that open more experiences—social-haptic communication over a distance, the ability to engage with items interactively and interactively and interrogate objects to offer more interactive tactile exploration. New devices or technologies should emphasise flexibility and interaction, enabling individuals to assemble their own solutions to their individual situations.

## Data Availability

No data are available.
